# Survival and predictors of mortality among severe acute malnourished under-five children admitted at Felege-Hiwot comprehensive specialized hospital, northwest, Ethiopia: a retrospective cohort study

**DOI:** 10.1186/s12887-021-02651-x

**Published:** 2021-04-16

**Authors:** Amare Kassaw, Desalegne Amare, Minyichil Birhanu, Aragaw Tesfaw, Shegaw Zeleke, Getachew Arage, Demewoz Kefale

**Affiliations:** 1 Department of Pediatrics and Child Health Nursing, College of Health Sciences, Debre Tabor University, P.O.Box:272, Debre Tabor, Ethiopia; 2 Department of Pediatrics and Child Health Nursing, College of Medicine and Health Sciences, school of Health Sciences, Bahir Dar University, Bahir Dar, Ethiopia; 3 Department of Public Health, College of Health Sciences, Debre Tabor University, Debre Tabor, Ethiopia; 4 Department of Adult Health Nursing, College of Health Sciences, Debre Tabor University, Debre Tabor, Ethiopia

**Keywords:** Mortality, Severe acute malnutrition, Survival, Under-five children

## Abstract

**Background:**

Malnutrition is still a global public health problem contributing for under-five morbidity and mortality. The case is similar in Ethiopia in which severe acute malnutrition is the major contributor to mortality being an underlying cause for nearly 45% of under-five deaths. However, there is no recent evidence that shows the time to death and public health importance of oxygen saturation and chest in drawing in the study area. Therefore, estimated time to death and its predictors can provide an input for program planners and decision-makers.

**Methods:**

A facility -based retrospective cohort study was conducted among 488 severe acute malnourished under-five children admitted from the 1st of January 2016 to the 30th of December 2019. The study participants were selected by using simple random sampling technique. Data were entered in to Epi-Data version 3.1 and exported to STATA version15 statistical software for further analysis. The Kaplan Meier was used to estimate cumulative survival probability and a log-rank test was used to compare the survival time between different categories of explanatory variables. The Cox-proportional hazard regression model was fitted to identify predictors of mortality. *P*-value< 0.05 was used to declare statistical significance.

**Results:**

Out of the total 488 randomly selected charts of children with severe acute malnutrition, 476 records were included in the final analysis. A total of 54(11.34%) children died with an incidence rate of 9.1death /1000 person- days. Failed appetite test (AHR: 2.4; 95%CI: 1.26, 4.67), altered consciousness level at admission (AHR: 2.4; 95%CI: 1.08, 4.67), oxygen saturation below 90% (AHR: 3.3; 95%CI: 1.40, 7.87), edema (AHR 2.9; 95%CI: 1.45, 5.66) and HIV infection (AHR: 2.8; 95%CI: 1.24, 6.36) were predictors of mortality for children diagnosed with severe acute malnutrition.

**Conclusion:**

The overall survival status of severe acute malnourished children was low as compared to national sphere standards and previous reports in the literature. The major predictors of mortality were oxygen saturation below 90%, altered consciousness, HIV infection, edema and failed appetite test. Therefore, early screening of complications, close follow up and regular monitoring of sever acute malnourished children might improve child survival rate.

## Background

Malnutrition refers to all deviations from adequate nutrition and jeopardizes children’s survival, health, growth and development. It exists in two forms; over nutrition and undernutrition of macronutrients and micronutrients [1, 2]. In the developing world undernutrition or protein-energy malnutrition is the common problem in acute or chronic form that profoundly affects children’s survival [3]. Undernutrition comprises of wasting, stunting and underweight [4]. Severe acute malnutrition (wasting) is defined as very low weight for length/height (WFL/WFH) below − 3 z scores of the median World Health Organization (WHO) growth standards or less than 70% of the median National Center for Health Statistics standard or the presence of nutritional edema [5].

Severe acute malnutrition remains the major cause of child morbidity and mortality worldwide [1, 2]. In 2017, 51 million children under-5 years were wasted and 16 million were severely wasted globally [6]. Annually about 7.6 million deaths among children under-5 years of age [7], from these 35% deaths were nutritional related and 4.4% of deaths were specifically attributable to severe wasting [8]. The status of the world’s children 2019 report showed that nearly half of all death in children under-5 years attributed to undernutrition; it puts children at greater risk of dying from common infections, increases the frequency, severity of such infections and delays recovery time. Children with severe acute malnutrition are nearly 12 times more likely to die than healthy children [3].

Globally, it is estimated that around 1 to 2 million children die every year due to severe acute malnutrition. Most of them live in South Asia and Sub-Saharan Africa [9, 10]. The mortality rate due to SAM in Sub-Saharan Africa ranges from 9.8 to 46% [11–14]. Evidences indicated that the likely cause of high mortality rate could be HIV infection [11, 15], lack of appetite, chest in drawing [14], co-morbidities and altered consciousness at admission [13, 16].

Despite Ethiopia is planning and implementing National Newborn and Child Survival Strategy to reduce under-five mortality from 64/1000 to 29/1000 by 2019/2020 [17], 2019 Mini Ethiopia Demographic and Health survey report indicated that under-five mortality is 55/1000 [18]. Malnutrition is the major contributor to mortality in Ethiopia being an underlying cause for nearly 45% of under-five deaths [17].

Even though Ethiopia uses a standard protocol for the management of severe acute malnutrition, it is the third leading cause of mortality in under-5 years old children and more than one-fourth of deaths are occurring during hospital admission [4, 19].

Several studies conducted in Ethiopia showed that HIV infection, shock, anemia and TB were significant predictors of mortality and low survival [4, 13, 20, 21]. However, these studies did not consider factors like low oxygen saturation, chest in drawing and fast breathing which have significant effect on child mortality admitted with SAM [14, 22]. Determining the magnitude and predictor of morality is a vital issue. Therefore, this study can contribute to the provision of data that is essential in planning, prioritize budgeting and staff training that improves the management system of public health settings and reduce child mortality.

## Methods

### Study area and period

This study was conducted at Felege Hiwot Comprehensive Specialized Hospital. The hospital is found in Bahir Dar city which is the capital city of Amhara regional state and located approximately 565 km Northwest of Addis Ababa, the capital city of Ethiopia. The governmental health institutions in the city include one Specialized Comprehensive Hospital, teaching hospital, primary hospital and 10 health centers. The pediatric ward has 47 beds and an emergency unit with 10 resuscitation beds. It has a total of 45 health professionals (4 pediatricians, 8 general practitioners and 33 nurses). The study was conducted from February 27 to March 15, 2020.

### Study design and participant characteristics

A facility-based retrospective follow up study was conducted. All under-five children diagnosed with severe acute malnutrition who were admitted during January 2016–December 2019 at FHCSH included in the study.

### Sample size determination and sampling procedure

The sample size was calculated by using Epi- info Version 7 statistical software with the assumptions of 95% CI, 5% margin of error, 80% power, exposed to unexposed ratio 1:1, 10% for incomplete records and an important variable of the study (passed appetite test) (P1):4% and adjusted hazard ratio (AHR:2.75) [23]. The total under-5 years aged SAM children admitted to TFU from January 2016—December 2019 at FHCSH were 1340. Unique chart numbers of SAM children were written on Microsoft excel. Then Simple random sampling technique was applied to select individual records using computer generation method and a random sample of 488 SAM children were selected.

### Data collection tools and procedure

The data were collected by three trained professional nurses through document review with a structured checklist. The checklists were consisted socio-demographic, co-morbidities, feeding and treatment related factors which are adopted and modified from different related studies [4, 21, 24], reviewing the medical charts and severe acute malnutrition management protocol guideline. The starting point for retrospective follow-up study was the time from first admission date and the endpoint was date of death and censored. The survival status of patients was obtained from the medical records. Survival time was calculated as the time between the dates of admission to the date of death, censored. All medical records of severe acute malnourished under-five children that fulfill the inclusion criteria who were admitted to TFU at FHCSH from the first of January, 2016 to December 30th, 2019 were retrospectively reviewed.

### Data quality assurance

Data quality was assured by designing proper data extraction tools. The adopted data extraction tools were evaluated by experienced researchers. A pretest was done on 5% of the sample size with a structured checklist at FHCSH before starting the actual study period and necessary corrections were done**.** After the pretest**,** unrecorded variables were reduced from the data extraction tools and others were arranged as per medical records. Training was provided for data collectors and supervisor before data collection and there was close follow up of data collectors by supervisor and the principal investigator. The collected data were checked out for completeness, accuracy and clarity by the principal investigator and supervisor.

### Operational definition of terms

#### Censored

Severe acute malnourished under-five children at TFU with predictors but recovered and discharged to home, discharged against medical advice or transfer out to other health institutions without knowing the outcome [4].

#### Survival status

Is the final outcome of severe acute malnourished children either death or censored.

#### Event

Death.

#### Incomplete records

If date of admission, date of discharge and final outcome not recorded on the participants chart.

#### Co-morbidities

Medical complications that will occur as a result of severe acute malnutrition (diarrhea, tuberculosis, pneumonia, CHF, anemia, shock, and hypoglycemia).

#### Survival time

Measures the follow-up of time from a defined starting point/from admission of under-five children diagnosed with SAM to TFU up to the occurrence of the event.

#### Follow up time

From the time of admission of under-five children diagnosed with SAM until either an event or censorship occurs**.**

#### Length of stay

The number of days the child stayed in the hospital from admission until death or censoring [4].

### Data processing and analysis

Before analysis, the collected data were cleaned, coded and entered in to Epi-Data Version 3.1 and exported to STATA 15 statistical software. Multiple imputations were done for handling missing values. The continuous data, depending on the distribution, were described either in mean and standard deviation or median. Frequency distribution was used for categorical data.

Finally, the outcome of each participant was dichotomized into censored or death. Incidence density rate (IDR) was calculated for the entire study period. Kaplan Meir was used to estimate mean survival time and cumulative survival probability and log-rank test was used to compare the survival time between different categories of explanatory variables.

Before running Cox proportional hazard regression model multi-collinearity was checked using variance inflation factor (VIF) and with pair-wise correlation. Residuals also were checked using goodness-of-fit test by Cox Snell residuals, which was satisfied the model test.

Proportional Hazard assumption was checked using the Schoenfeld residual statistical test.

Bi-variable Cox-proportional hazard regression model was fitted for each explanatory variable. Variables having *p*-value < 0.2 in the bi-variable analysis was fitted to the multivariable Cox-proportional hazards regression model. Hazard ratio with 95% confidence interval and p-value < 0.05 was used to measure the strength of association and to identify statistical significant predictors.

### Ethical consideration and consent to participate

Ethical clearance was obtained from Bahir Dar University, College of Medicine and Health Sciences, Ethical Clearance Review Committee namely (Taddesse Dagget, Yinager Workineh, Amlaku Mulat, Abirham Belachew and Chalachew Genet) with protocol number 0051/2020 and Institutional Review Board (IRB) decision number 002.Then; official letter was submitted to FHCSH. The data were collected after getting consent from the hospital manager; since it was retrospective chart review, direct informed consent from the patient was not required. This study didn’t inflict or exposes children to unnecessary risk as a result of reviewing their medical records. Confidentiality of data was kept at all levels of the study and the data was not used for other purposes other than for this study. The study was conducted in accordance with the Declaration of Helsinki relevant guidelines and regulations.

## Results

### Socio-demographic characteristics

Out of the total 488 randomly selected charts of SAM children, 476(97.5%) records were fulfilling enrollment criteria in the final analysis; and the remaining 12(2.5%) were excluded (8 incomplete data and 4 charts were lost during data collection). Out of them more than half 245(51.5%) of the children enrolled in the study were males. Two-third of the participants 321(67.4%) came from rural area. The median age of children was 14 months with (IQR = 19) and majority of 345(72.5%) were aged less than 24 months (Table 1).

**Table 1:** Socio-demographic characteristics and nutritional status of SAM children admitted at FHCSH from 2016 to 2019, Northwest Ethiopia, 2020 (*n* = 476)

Variables	Categories	Frequency n (%)
Age in month	< 24	345 (72.5)
	≥24	131 (27.5)
Sex of the child	Male	245 (51.5)
	Female	231 (48.5)
Residence	Urban	155 (32.6)
	Rural	321 (67.4)
Type of SAM (*N* = 472)	Marasmus	278 (58.9)
	Kwashiorkor	130 (27.5)
	marasmus-kwash	64 (13.6)
WHZ (*n* = 470)	Z-score ≥ −3	182 (38.7)
	Z-score < − 3	288 (61.3)
HAZ (*n* = 471)	Z-score < −3	246 (52.2)
	Z-score ≥ −3	225 (47.8)
WAZ (*n* = 470)	Z-score < −3	150 (31.9)
	Z-score ≥ −3	320 (68.1)

### Routine medication and feeding-related characteristics

Four hundred sixty-five (97.6%) children had taken routine antibiotics, 337(70.8%) and 206 (43.2%) had received vitamin A and Zink supplementation. About three fourth of the children had given nutritional therapy (F-75 and F-100) and 121(25.7%) failed appetite test (Table 2).

**Table 2:** Treatment and feeding patterns of SAM children admitted at FHCSH from 2016 to 2019, Northwest Ethiopia, 2020 (*n* = 476)

Variables	Categories	Frequency n (%)
Routine antibiotics	Yes	465 (97.7)
	No	11 (2.3)
Intake of F-100 (*n* = 474)	Yes	357 (75.3)
	No	117 (24.7)
Intake of F-75 (475)	Yes	353 (74.3)
	No	122 (25.7)
Folic acid given	Yes	363 (76.3)
	No	113 (23.7)
Vitamin A given	Yes	337 (70.8)
	No	139 (29.2)
Nasogastric feeding (*n* = 472)	Yes	144 (30.5)
	No	328 (69.5)
Blood transfusion (*n* = 474)	Yes	57 (12.0)
	No	417 (88.0)
Appetite test (*n* = 471)	Failed	121 (25.7)
	Passed	350 (74.3)

### Clinical conditions and major co- morbidities

Most of the children were in critical condition at the time of admission. The common co- morbidities identified during admission were anemia (43.8%), diarrhea (40.9%), pneumonia (40.7%) pulmonary TB (13.0%) and CHF (12.5%) (Table 3).

**Table 3:** Clinical conditions and major co-morbidities of SAM children admitted at FHCSH from 2016 to 2019, Northwest Ethiopia, 2020 (*n* = 476)

Variables	Categories	Frequency n (%)
HIV/AIDS (*n* = 376)	Reactive	31 (8.3)
	Non-reactive	345 (91.7)
Pneumonia (*n* = 472)	Yes	192 (40.7)
	No	280 (59.3)
Pulmonary TB (*n* = 471)	Yes	61 (13.0)
	No	410 (87.0)
CHF (*n* = 473)	Yes	59 (12.5)
	No	414 (87.5)
UTI (*n* = 475)	Yes	35 (7.4)
	No	437 (92.6)
Hypoglycemia (*n* = 471)	Yes	45 (9.6)
	No	426 (90.4)
Shock (*n* = 471)	Yes	16 (3.4)
	No	455 (96.6)
Chest in drawing (*n* = 472)	Yes	107 (22.7)
	No	365 (77.3)
Oxygen saturation (*n* = 472)	< 90%	181 (38.4)
	≥90%	291 (61.6)
Diarrhea (*n* = 472)	Yes	192 (40.7)
	No	280 (59.3)
Anemia (hgb < 11 mg/dl)	Yes	206 (43.8)
	No	264 (56.2)
Fast breathing (*n* = 472)	Yes	144 (30.51)
	No	328 (69.5)
Admission Pulse rate	Altered	273 (57.4)
	Normal	203 (42.6)
Admission respiratory rate	Altered	214 (45.0)
	Normal	262 (55.0)
Status at admission (*n* = 472)	Altered	27 (5.7)
	Conscious	445 (94.3)

### Survival status of severe acute malnourished children

A total of 476 were followed for different periods: a minimum of 2 and a maximum of 46 days and overall median hospital stay were 11 days. Of 476 children whose recorded were reviewed, 54(11.3%) with (95% CI: 8.40, 14.30) died and 422(88.7%) censored (among them 324(68.1%) recovered 14(2.9%) were referred, 36(7.6%) defaulted and 48 (10.1%) were against medical advice at the end of the study period. The study participants were followed for 5936 person –day with an incidence rate of 9.1death per 1000 person- day observation (95% CI: 6.97, 11.88).

This study also showed that the incidence of mortality in HIV infected children was higher than HIV uninfected with an incidence rate of 25.7%/1000(95% CI: 15.00, 44.00). The incidence of mortality in SAM children who were impaired consciousness during admission was also higher compared to conscious with an incidence rate of 44.4%/1000 (95% CI: 27.00, 72.00).

### Overall survival function

**Fig. 1:**
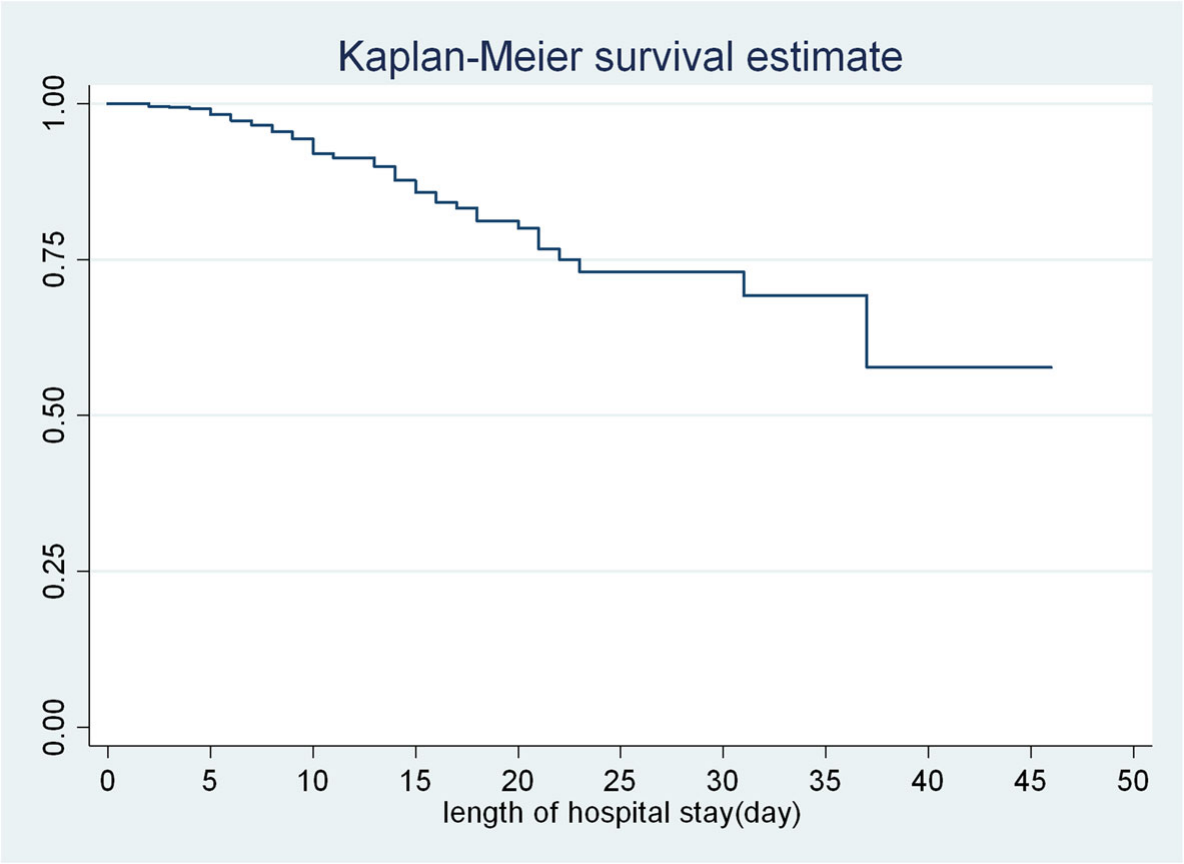
Overall Kaplan-Meier survival estimate of SAM children admitted to TFU of FHCSH, from 2016 to 2019, Northwest Ethiopia, 2020

The survival probability of severe acute malnourished children was estimated using Kaplan-Meier estimate. The survival probability in the second day of admission was high almost (99.6%) with a standard error of 0.003(95% CI: 98, 99.9). At the 12th day of hospital stay the survival probability of SAM children was also found to be 90.9% with a standard error of 0.0156(95% CI: 87.00, 94.00), from 20 to 25 days of hospital stay the probability of surviving was 72.8% with a standard error of 0.0424(95%CI: 63.00, 80.00) and at the end of 45 days the overall survival probability was 59.2% with standard error 0.099(95% CI: 38.00, 76.00) (Fig. 1).

### Survival function and comparison of survivorship functions

**Fig. 2:**
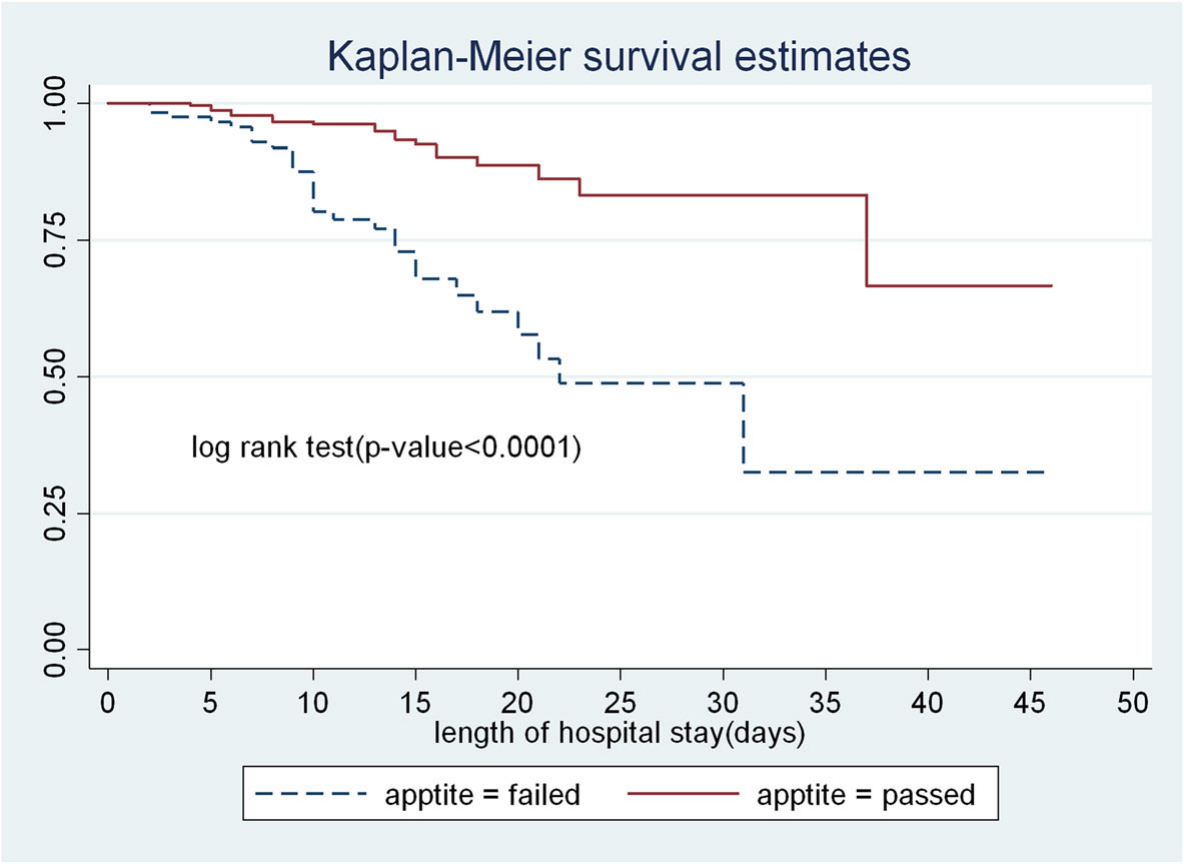
The Kaplan-Meier survival curves compare the survival time of SAM children admitted with categories of appetite test to TFU of FHCSH, from 2016 to 2019, North West Ethiopia, 2020

In this study, SAM children who failed appetite test at admission had lower survival time compared to those who passed appetite test. At the 35 days of hospital stays the cumulative survival probability of SAM children who failed appetite test were 32.4% as compared to those passed appetite test (84.1%) (Fig. 2).

**Fig. 3:**
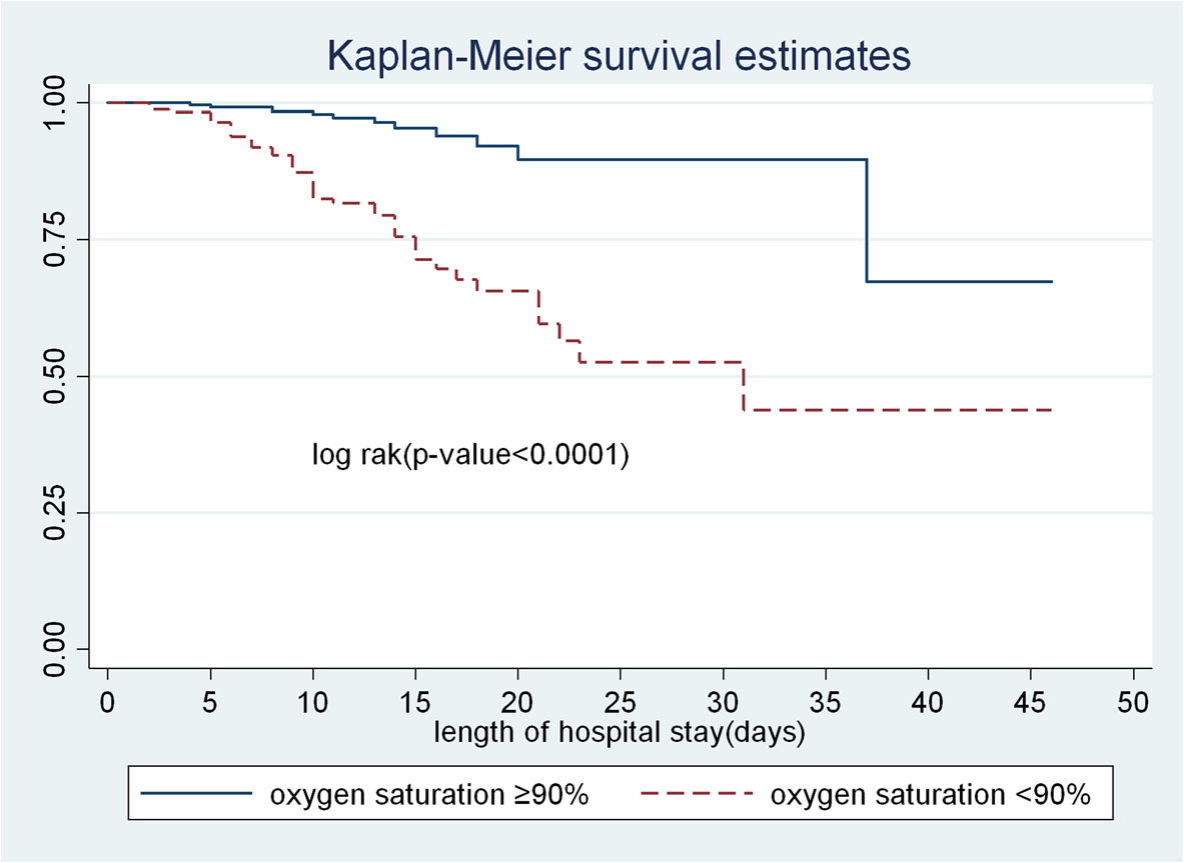
The Kaplan-Meier survival curves compare the survival time of SAM children admitted with categories of oxygen saturation level to TFU of FHCSH, from 2016 to 2019, Northwest Ethiopia, 2020

The survival time of SAM children with oxygen saturation less than 90% was also lower than those with saturation greater than 90%. The overall survival at the end of the follow up period was 44.2% for those who had oxygen saturation < 90% compared to their counterparts, 70.4% (Fig. 3).

**Fig. 4:**
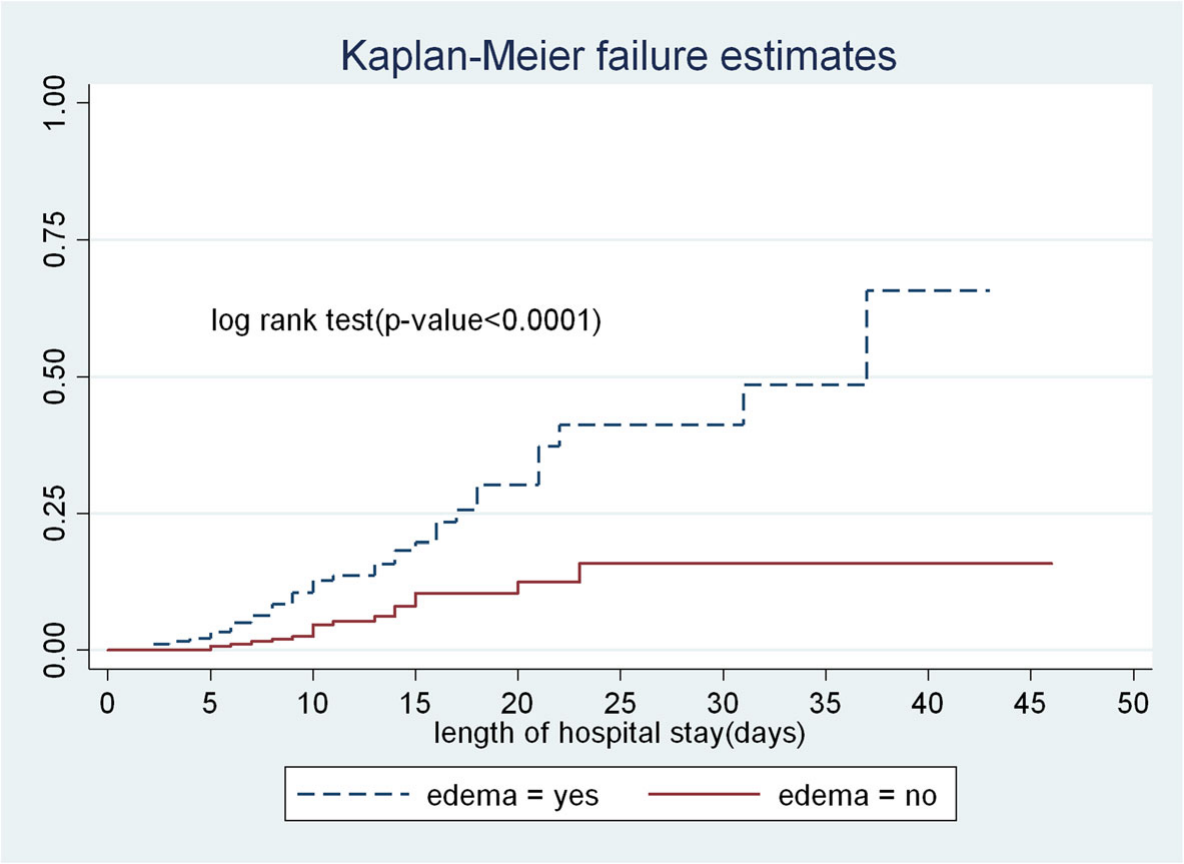
The Kaplan-Meier hazard curves compare hazard time of edematous children with Categories of non-edematous children, to TFU of FHCSH, from 2016 to 2019, Northwest Ethiopia, 2020

The survival time of non-edematous children was longer than edematous children. At the end of the study, the hazard of death for those edematous children was 61.5% as compared to non-edematous children 16.9%(Fig. 4).

### Predictors of mortality among severe acute malnourished children

The relationship between the baseline variables and the risk of mortality was analyzed using bi-variable Cox proportional hazard regression model. From the bi-variable analysis, HIV/AIDS, pneumonia, TB, edema, Nasogastric tube feeding, chest in drawing, type of SAM, oxygen saturation below 90%, altered pulse rate and respiratory rate (at admission), failed appetite test, anemia (hgb < 11 mg/dl), intake of F-100, WHZ, HAZ, impaired consciousness at admission are predictors of mortality in SAM children. To identify independent predictors of mortality, multivariable Cox regression was performed for variables significant in bi-variable analysis. Only six variables oxygen saturation below 90%, impaired consciousness at admission, intake of F-100, HIV/AIDS, edema and failed appetite test were significant predictors in the multivariable analysis.

The results of multivariable analysis showed that children who failed appetite test at admission were 2.44 hazard of death as compared to who have passed appetite test (AHR: 2.45; 95%CI: 1.28, 4.69). The risk of mortality of children who impaired consciousness level at admission were 2.25 times as compared to those conscious at admission (AHR:2.25; 95%CI: 1.08,4.68) (Table 4).

**Table 4:** Results of bi-variable and multivariable Cox regression analysis of SAM children admitted at FHCSH from 2016 to 2019, Northwest Ethiopia, 2020

Variables		Survival Status		CHR (95% CI)	AHR (95%CI)
		Event (%)	Censored (%)		
HIV/AIDS	Reactive	13 (41.9)	18 (58.1)	2.83 (1.49,5.36)	2.80 (1.24, 6.34)*
	Non-Reactive	37 (10.7)	308 (89.3)	1	1
TB	Yes	15 (24.6)	46 (75.4)	2.24 (1.23,4.09)	1.84(.88,3.85)
	No	39 (9.5)	371 (90.5)	1	1
Pneumonia	Yes	33 (17.2)	159 (82.8)	2.45 (1.41,4.24)	0.95 (0.45, 2.00)
	No	21 (7.5)	259 (92.5)	1	1
oxygen saturation	< 90%	40 (22.0)	142 (78.0)	5.85 (3.08,11.1)	3.32 (1.40,7.88)*
	≥90%	14 (4.8)	276 (95.2)	1	1
Appetite test	Failed	31 (25.0)	93 (75.0)	4.2 (2.45,7.33)	2.45 (1.28, 4.69)*
	Passed	22 (6.3)	325 (93.7)	1	1
WHZ	Z-score < −3	40 (13.9)	248 (86.1)	2.09 (1.13,3.85)	1.52 (0.69, 3.41)
	Z-score ≥ − 3	14 (7.7)	168 (92.3)	1	1
IntakeofF-100	No	23 (19.5)	95 (80.5)	2.96 (1.71,5.10)	2.63 (1.35,5.12)*
	Yes	31 (8.7)	327 (91.3)	1	1
NGT feeding	Yes	26 (17.8)	120 (82.2)	2.34 (1.37,4.00)	1.06 (0.55,2.03)
	No	28 (8.5)	302 (91.5)	1	1
Edema	Yes	36 (19.2)	151 (80.8)	3.05 (1.73,5.37)	2.85 (1.44,5.64)*
	No	18 (6.2)	271 (93.8)	1	1
Admission status	Impaired	16 (51.6)	15 (48.4)	6.66 (3.69,12.0)	2.25 (1.08,4.68)*
	Conscious	38 (8.54)	407 (91.46)	1	1
Admission pulse rate	Altered	41 (15.0)	232 (85.0)	2.23 (1.19,4.16)	1.02 (0.42,2.46)
	Normal	13 (6.4)	190 (93.6)	1	1
Admission respiratory rate	Altered	38 (17.8)	176 (82.2)	2.98 (1.66,5.35)	0.94 (0.38, 2.29)
	Normal	16 (6.1)	246 (93.9)	1	1
HAZ	Z-score < −3	33 (14.7)	192 (85.3)	2.01 (1.16,3.48)	1.60 (0.75,3.42)
	Z-score ≥ − 3	21 (8.5)	225 (91.5)	1	1
Chest indrawing	Yes	26 (24.3)	81 (75.7)	3.05 (1.79,5.22)	1.82 (0.86, 3.87)
	No	28 (7.7)	337 (92.3)	1	1
Anemia	Yes	33 (15.5)	180 (84.5)	1.67(.96, 2.90)	1.78 (0.97,3.27)
	No	21 (8.0)	242 (92.0)	1	1

NB. * significant (*p*-value less than 0.05)

## Discussion

This study revealed that the overall mortality of severe acute malnourished children was 11.34% with (955% CI: 8.40, 14.30) during the follow-up period. This finding is consistent with studies done, in Uganda [14], in Egypt [25] and studies conducted in Ethiopia, Jimma university [16], Gondar university [4] and Gedio Zone [26]. On the other hand, this finding is lower than a study done in South Africa [12] and other study in Uganda [15]. The discrepancy might be due to high proportion of HIV positive cases, in South Africa, 43% were HIV infected and this in turn increases the mortality due to the complexity of the management and opportunist infections. Similarly, the result is lower than the study conducted in Ethiopia, Sekota hospital [21], and Zewditu hospital [27]. The difference can be due to differences in management team and supplies, the difference in the study period as there were changes in treatment modality and might be due to an increasing number of co-morbidity and severity of cases. However, the finding of this study was higher than a study in general hospital of Tigray [13]. The possible justification of this discrepancy could be an increased complicated cases with co-morbidities like HIV/AIDS in our study which increased risk of death [12].

The overall incidence of mortality was found to be 9.1death per 1000 person- days (95% CI: 6.97–11.88). This finding was in agreement with a study in Dilchora referral hospital 7.5 deaths/1000 person-days [23] and Gondar university 10.4 deaths /1000 person-days [4]. On the contrary, the result was lower than a study done in Uganda 24 deaths/1000 person- days. This might be due to the difference in methodology which was a prospective cohort in the previous study. As a result, it included patients discharge following against medical advice and prior co-morbidities, for example in Uganda of the children were HIV-infected [15].

The survival probability was also found to be 90.9 and 72.8%, at the 12th and 20-25th days respectively. The overall cumulative survival probability at the end of 45th days was 59.2%. As result, median survival time could not determine. This study also indicated that the average length of stay in the hospital was 12.5 days. This is in congruent with a study in Gondar University 12 days [4], Dilchora Hospital 10 days [23], and international minimum (SPHERE) standard average length of wait that sets not exceed 30 days [28].

In the current study, SAM patients with oxygen saturation below 90% were more hazard of death as compared to those with saturation above 90%. This result is supported by a finding in Uganda [14]. This might be due to low oxygen saturation in the body causing respiratory failure, interference in brain and heart functions in SAM children [29].

SAM children presented with altered conscious level at admission were more hazard of death as compared to those conscious at admission. This was congruent with the finding in Jimma, Tigray and Kenya [13, 16, 30]. This might be because of a child with impaired conscious level increases the management complexity that is prone the children to nasogastric feeding, intravenous medication and fluid which were further reducing survival time [2, 5].

This study also revealed that not intake of F-100 was a significant predictor of mortality in SAM children. This is comparable with a study in Dilchora Hospital. The possible justification might be nutritional therapies (therapeutic feeding) are important in SAM children in reduction adaptation to maintain metabolic rate [5].

This study investigated that HIV infected children were 2.8 folds hazard of death as compared to HIV- uninfected children. This was supported by a study conducted in Uganda Africa, Malawi and Zambia [11, 12, 14, 31]. The result also in agreement with a study in Ethiopia [23]. The possible reason could be HIV/AIDS together with malnutrition is particularly fatal because it increases management complexity and opportunistic infection which could impair feeding of SAM children [2, 3].

Failed appetite test at admission was 2.45 times hazard of death as compared to those passed appetite test and this result was comparable with similar studies [14, 23]. The possible scientific justification might be, reduction in appetite is the sign of severe metabolic malnutrition and it is mainly metabolic malnutrition that causes death. A poor appetite means that the child has a significant infection or a major metabolic abnormality such as liver dysfunction, electrolyte imbalance and cell membrane damage. These are the patients at immediate risk of death [2].

In the current study Children presented with edema at admission were 2.9 times risk to die than non-edematous children. This finding is supported by a study done in Zambia [32]. The possible justification could be due to edematous patients exhibit reduced ability to recover fluids, immune system failure, low lipid absorption and the liver is enlarged with fat so that, less ready to make glucose and exposed the child for hypoglycemia [1].

Although the current study did not show any significant association between severe acute malnutrition children with pneumonia, shock, TB, type of SAM, diarrhea, anemia, CHF and mortality, other investigations conducted in Zambia and South Africa [11, 12] and in Ethiopia [16, 21, 23, 26] showed that there was a significant association with child mortality. This discrepancy probably because of difference in sample size or might be the difference in the study period as there were changes in treatment modality and updating of professionals in standard training and regular supervision. The other possible justification could be strictly using the national management protocol, for example, TB and SAM management guidelines.

### Limitation of the study

Since the data were retrospectively collected from patients’ medical records, some important variables were excluded such as, parents’ socio-demographic characteristics and the others may not recorded at all. Therefore, those threats might influence the outcome.

## Conclusion

In this study, the overall survival status of severe acute malnourished children was low and incidence of mortality was high compared to international minimum (SPHERE) standard and previous reports in the literature. The major predictor of mortality were oxygen saturation below 90%, not intake of F-100, HIV infection, edema, altered conscious level at admission and failed appetite test. Therefore, the health care workers should closely screen and give follow- up for severe acute malnourished children particularly those with identified predictors of death. Programmed monitoring and supervision concerning with national SAM standard management protocol is also highly recommended. Additionally longitudinal prospective follow up studies are recommended to identify additional socio-demographic factors, environmental factors and biochemical tests.

## Data Availability

Data will be available upon request from the corresponding author.

## Abbreviations

AIDS: Acquired Immune Deficiency syndrome
AHR: Adjusted Hazard Ratio
CI: Confidence Interval
HAZ: Height for Age Z-score
HIV: Human Immunodeficiency Virus
HR: Hazard Ratio
SAM: Severe Acute Malnutrition
WAZ: Weight for Age Z-score
WHZ: Weight for Height Z-score
IQR: Inter Quartile Range
WHO: World Health Organization
TFU: Therapeutic Feeding Unit
